# Oral Misoprostol alone versus oral misoprostol followed by oxytocin for labour induction in women with hypertension in pregnancy (MOLI): protocol for a randomised controlled trial

**DOI:** 10.1186/s12884-021-04009-8

**Published:** 2021-07-29

**Authors:** Hillary Bracken, Kate Lightly, Shuchita Mundle, Robbie Kerr, Brian Faragher, Thomas Easterling, Simon Leigh, Mark Turner, Zarko Alfirevic, Beverly Winikoff, Andrew Weeks

**Affiliations:** 1 Gynuity Health Projects, 220 East 42nd Street, Suite 710, New York, NY 10017 USA; 2 Department of Women’s and Children’s Health, Liverpool Women’s Hospital, University of Liverpool, Crown Street, Liverpool, L8 7SS UK; 3 Obstetrics and Gynecology, All India Institute of Medical Sciences, Plot no 2, Sector 20, Mihan Nagpur, 441108 India; 4 Fetal Medicine, St Michael’s Hospital, Marlborough Street, BS1 3NU Bristol, UK; 5 Medical Statistics, LSTM Clinical Group, Liverpool School of Tropical Medicine, Pembroke Place, Liverpool, L3 5QA UK; 6 Department of Obstetrics and Gynecology, University of Washington, Seattle, WA 98195 USA; 7 Nexus Clinical Analytics, Ltd, 15 Glencroft, Euxton, PR7 6BX Lancashire UK

**Keywords:** Pre-eclampsia, Induction of labour, Misoprostol, Oxytocin, Augmentation of labour, Randomized controlled trial, Study protocol

## Abstract

**Background:**

Every year approximately 30,000 women die from hypertensive disease in pregnancy. Magnesium sulphate and anti-hypertensives reduce morbidity, but delivery is the only cure. Low dose oral misoprostol, a prostaglandin E1 analogue, is a highly effective method for labour induction. Usually, once active labour has commenced, the misoprostol is replaced with an intravenous oxytocin infusion if ongoing stimulation is required. However, some studies have shown that oral misoprostol can be continued into active labour, a simpler and potentially more acceptable protocol for women. To date, these two protocols have never been directly compared.

**Methods:**

This pragmatic, open-label, randomised trial will compare a misoprostol alone labour induction protocol with the standard misoprostol plus oxytocin protocol in three Indian hospitals. The study will recruit 520 pregnant women being induced for hypertensive disease in pregnancy and requiring augmentation after membrane rupture. Participants will be randomised to receive either further oral misoprostol 25mcg every 2 h, or titrated intravenous oxytocin. The primary outcome will be caesarean birth. Secondary outcomes will assess the efficacy of the induction process, maternal and fetal/neonatal complications and patient acceptability. This protocol (version 1.04) adheres to the SPIRIT checklist. A cost-effectiveness analysis, situational analysis and formal qualitative assessment of women’s experience are also planned.

**Discussion:**

Avoiding oxytocin and continuing low dose misoprostol into active labour may have a number of benefits for both women and the health care system. Misoprostol is heat stable, oral medication and thus easy to store, transport and administer; qualities particularly desirable in low resource settings. An oral medication protocol requires less equipment (e.g. electronic infusion pumps) and may free up health care providers to assist with other aspects of the woman’s care. The simplicity of the protocol may also help to reduce human errors associated with the delivery of intravenous infusions. Finally, women may prefer to be mobile during labour and not restricted by an intravenous infusion. There is a need, therefore, to assess whether augmentation using oral misoprostol is superior clinically and economically to the standard protocol of intravenous oxytocin.

**Trial registration:**

Clinical Trials.gov, NCT03749902, registered on 21^st^ Nov 2018.

## Background

Hypertensive disease in pregnancy is a major cause of the 300,000 maternal deaths that occur every year [1]. In South Asia alone, hypertensive disease in pregnancy is responsible for 10,000 deaths annually [2]. Much of this burden could be prevented by timely and effective delivery—the only curative intervention in pre-eclampsia.

Misoprostol, a prostaglandin E1 analogue, given in low doses orally is a highly effective method for labour induction in low resource settings. Oral administration of 25 µg every 2 h received a strong recommendation by WHO in 2011 [3]. The Cochrane review of oral misoprostol found that in low doses oral misoprostol is at least as effective as the commonly used vaginal dinoprostone gel [4]. Misoprostol is also heat stable and less than 1% of the cost of dinoprostone gel. The Cochrane review concluded that “low-dose oral misoprostol probably has many benefits over other methods for labour induction.” and that “a starting dose of 25 μg may offer a good balance of efficacy and safety.” A recent network meta-analysis of all prostaglandins for labour induction supported these conclusions, finding that oral misoprostol solution (< 50mcg) was the safest in terms of risk of caesarean section [5].

Until recently, misoprostol was only available in 100 and 200mcg tablets (Cytotec®) which had to be either cut or dissolved in water to obtain the 25mcg doses [6–8]. However, low cost, high quality 25mcg misoprostol tablets have now become available, removing many of the logistical barriers to its use.

Standard practice for induction of labour is to use a prostaglandin (dinoprostone or misoprostol) for cervical ripening. When the amniotic membranes rupture and active labour has commenced (cervical dilation of more than 3-4 cm), the provider stops administering the prostaglandin and an intravenous infusion of oxytocin is started if uterine stimulation is still required [9]. Every 30 min the infusion is increased to stimulate uterine contractions sufficient to progress labour, but not so much as to cause hyperstimulation and consequent fetal hypoxia.

However, avoiding oxytocin and continuing low dose misoprostol into active labour may have a number of benefits for women and the health care system. Misoprostol is heat stable and an oral medication and thus easy to store, transport and administer; all particularly desirable in low resource settings. The ease of administration of an oral medication may help to reduce human errors associated with the delivery of intravenous infusions and require less equipment (e.g., electronic infusion pumps). Moreover, the simplicity of the protocol, which eliminates the need to actively titrate an oxytocin infusion against contractions, may free up health care providers to assist with other aspects of a woman’s care. Finally, women may prefer to be mobile during labour and not restricted by an intravenous infusion. There is a need, therefore, to compare the safety, efficacy and acceptability of the two augmentation protocols. The primary objective of this trial is to assess whether augmentation using oral misoprostol is superior clinically and economically to the standard protocol of intravenous oxytocin.

## Methods

### Trial design

We will undertake a multi-centre parallel, superiority, open-label randomised trial in three publicly funded hospitals in India: Government Medical College Nagpur, Daga Memorial Women’s Hospital and Mahatma Gandhi Institute of Medical Sciences.

### Participants and recruitment

Research staff will invite 1,000 potentially eligible women, due to undergo induction of labour with oral misoprostol, to provide advance written informed consent in case they become eligible. We estimate that 1,000 women undergoing induction will be required in order to obtain a sample size of 520 for the randomised trial, as not all patients will require additional drugs after artificial rupture of membranes. The randomised study will enrol 520 pregnant women, ≥ 18 years of age, with a live fetus, who require induction because of pre-eclampsia or hypertension and who have undergone cervical ripening with oral misoprostol alone but require an additional augmentation agent after membrane rupture. Those women unable to give consent, with previous caesarean births, those who undergo cervical ripening with agents other than misoprostol (e.g. Foley catheter, other prostaglandins), with multiple pregnancy, a history of allergy to misoprostol or adequate uterine activity will be ineligible.

After a decision has been made to induce labour, a patient information leaflet about the study will be given to the participant to read, consider and discuss with her partner and/or family. This leaflet will be available in local languages. Trained research staff will be available to answer any questions that women and their families have about the study. All will be assured that their participation is voluntary and that their decision to participate or not participate will not affect their clinical care. If the woman decides that she would like to participate in the study, research staff will ask the participant to sign a consent form, prior to any induction agent being administered. Participants will be assigned a participant ID number and all study data will be maintained separate from any relevant identifiers to participant names. She would then commence cervical ripening with oral misoprostol, as per routine practice. Following cervical ripening, the amniotic membranes will be ruptured artificially. If further uterine stimulation is required 30 min after artificial rupture of membranes, and the patient wishes to continue participating in the study, she would be randomised into either the misoprostol or oxytocin group.

### Randomization and masking

After the patient orally confirms her wish to participate in the trial, the research staff will open a sequentially numbered, sealed, opaque envelope containing the participant’s group. Gynuity Health Projects will generate the envelopes using a randomisation code based on a computerised pseudo-random number generator. Randomisation will be stratified by centre, with randomly determined block sizes of 4, 6 or 8. Neither the participating providers nor the participants will be blinded to the group assignment due to the complexity of titrating two separate uterotonics simultaneously.

### Procedures

**Fig. 1:**
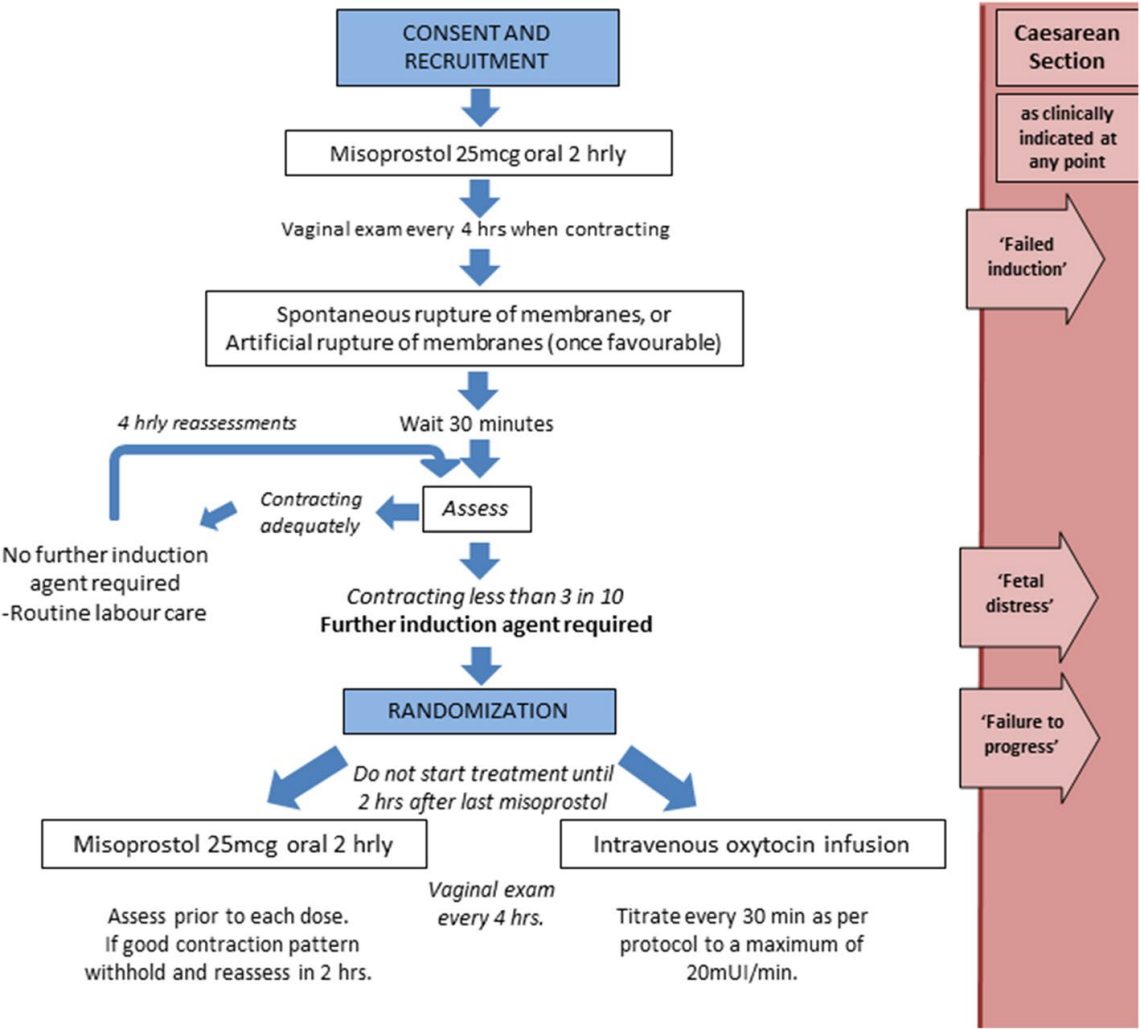
Randomized Trial Flowchart

The study flow is shown in Fig. 1. All women who consent to participate in the trial will undergo cervical ripening by the hospital staff who have received training in the study protocol. If the cervix is unfavourable, participants will receive oral misoprostol tablets (25 mcg) every 2 h for a maximum of 12 doses for cervical preparation. Once strong painful contractions (at least 3 in 10 min) have started, vaginal examination will be conducted. Contraction frequency will be assessed every 30 min and a vaginal examination done every 4 h to assess cervical dilation and Bishop score. Assessments may be conducted earlier if clinically indicated. The next dose of oral misoprostol will be omitted when moderate or strong contractions are occurring with a frequency of 3 in 10 min (i.e. 9 or more in the preceding 30 min). If the contractions become inadequate again, and the cervix is not yet 2 cm dilated then the misoprostol can be restarted at 25mcg every 2 h. If a clinical decision is made to use a mechanical method in addition to oral misoprostol for cervical preparation then the woman will no longer be eligible for the randomised trial. 'Artificial rupture of membranes’ (ARM) will be performed when the cervix is favourable (usually at 2 cm). No more cervical preparation doses of misoprostol will then be given. If spontaneous rupture of membranes occurs, then the cervical preparation doses of misoprostol will be stopped. If contractions are inadequate then further stimulation may be given as part of the randomised trial.

After membrane rupture, if contractions are continuing at 3 in 10 min (i.e., 9 or more in the preceding 30 min) or more and with progressive cervical change of at least 1 cm every 2 h then labour will continue without further augmentation. These participants will not be randomised unless the frequency of contractions reduce, or if cervical change becomes inadequate (< 1 cm every 2 h). However, if contractions reduce to less than 3 in 10 min (under 9 in 30 min) more than 30 min after ARM, then the next randomisation envelope will be opened by a research assistant, and the woman allocated to receive either continued oral misoprostol or an oxytocin infusion. The woman will also be eligible for randomisation if there is no progressive cervical change over this time, even if contracting well. If there is hyperstimulation (more than 5 contractions in 10 min), then any ongoing stimulation with misoprostol or oxytocin will be stopped until the hyperstimulation resolves.

Women randomised to receive misoprostol will receive an initial dose of oral misoprostol 25mcg. The next dose of oral misoprostol will be omitted if moderate or strong contractions are occurring at 3 in 10 min or more (i.e., 9 or more in the preceding 30 min). If contractions subsequently reduce to less than 3 in 10 min (i.e., less than 9 in 30 min) or become irregular or mild, then a further dose of oral misoprostol 25mcg will be administered, so long as 2 h have passed since the last dose. Oral misoprostol 25mcg may be continued every two hours in the event of inadequate uterine activity. In the event of inadequate progress, clinicians will be advised to give additional misoprostol unless the contractions are very strong and frequent. If there is suspicion of fetal distress due to excessive uterine activity then no additional misoprostol will be administered and terbutaline 250 µg will be given subcutaneously.

Women randomised to oxytocin will receive an oxytocin infusion through an intravenous cannula with an electronic infusion set. Five units of oxytocin will be injected in 500 mL of Ringer’s lactate, started at a rate of 2 mU/min, and increased every 30 min by 2 mU/min until there are three to four contractions every 10 min. The rate will be titrated to maintain that contraction frequency, with a maximum dose of 20 mU/min. If contractions exceed five contractions in 10 min then the oxytocin infusion will stopped and the infusion restarted at half the rate. If there is suspicion of fetal distress due to excessive uterine activity then the oxytocin infusion will be stopped. Terbutaline 250 µg subcutaneously will be given if excessive uterine activity persists.

For women in both groups, research staff will assess temperature and pulse every 2 h and the treating provider will perform a vaginal examination every 4 h to assess cervical dilation and progress. Research staff will document contraction strength and frequency every 30 min and with any change in the infusion rate. Initial fetal monitoring will be with a Fetal Pinnard’s stethoscope or fetal Doppler auscultation every 30 min as per hospital policy. Continuous electronic fetal monitoring will be commenced in the event of abnormalities detected on intermittent auscultation, but discontinued again if the fetal heart rate tracing is normal. Research staff will document the indication for and use of the cardiotocograph. Magnesium sulphate, steroids and anti-hypertensives will be given as per hospital protocols at provider discretion.

Research staff will ask all those recruited (whether eventually randomised or not) to complete an adapted version of the Mother-Generated Index (MGI) survey, the Mother Generated Birth Satisfaction Index (MGBSI) during the study [10, 11]. Research staff will administer a questionnaire at the time of recruitment to record the woman’s priorities and expectations for the induction process and labour. The research staff will administer a second questionnaire 24 h after the birth to assess the woman’s satisfaction with her pre-determined priorities, plus any other important issues. Together, the questionnaires will provide a quantitative satisfaction score as well as qualitative insights into the scoring. Research staff will also conduct a post-study interview with the woman 24 h after delivery and prior to discharge from the study hospital in order to document future preferences for induction of labour methods and overall acceptability of labour induction method.

During their time in the study, research assistants will enter participant’s non-identifiable study data directly into a password protected electronic case report form (REDCap, Vanderbilt University, Tennessee). Data on pregnancy and neonatal outcomes will also be collected from the hospital maternity records. The data will be stored on the handheld device until the device is synchronised through an encrypted link with the host server. Data will be stored on a password-protected secure server located in India. Only researchers from the study will have access to the data.

### Outcomes

The primary outcome will be caesarean birth, a core outcome used in the Cochrane Collaboration induction of labour generic protocol [12], as the induction of labour core outcome set [13] had not been published at the time of protocol development. A tble of assessments is shown in Table 1. The need for caesarean birth combines the efficacy of the induction (important when induction is being conducted for hypertension in pregnancy) with the safety of the induction process for mother and fetus. It is a particularly important outcome as caesarean birth poses a serious risk to maternal health in the context of the setting and hypertensive disease. Secondary outcomes will include measures of efficacy of the induction process (randomisation to birth interval; vaginal births within 12 and 24 h of randomisation; duration of hospital stay); additional outcomes to allow meta-analysis (start of induction to birth interval; cervix unchanged at 12 and 24 h after the start of cervical preparation; vaginal births within 12 and 24 h after the start of cervical preparation); maternal complications (uterine tachysystole (defined as > 5 contractions in 10 min), uterine hypertonus (defined as a single contraction lasting over 2 min); and hyperstimulation syndrome (tachysystole/hypertonus with associated fetal heart rate abnormalities); uterine rupture; instrumental vaginal delivery; severe hypertension and HELLP Syndrome; maternal vomiting, diarrhoea or fever; antibiotic use; postpartum haemorrhage (> 1,000 ml, or any bleeding leading to systolic blood pressure less than 90 mmHg or diastolic blood pressure less than 60 mmHg or blood transfusion)) and serious maternal complications (intensive care unit admission; septicaemia; pulmonary oedema; cerebral haemorrhage or oedema; renal failure; eclampsia and maternal death). Maternal satisfaction will be assessed using a ‘Mother Generated Birth Satisfaction Index.’ Health economic data will also be collected to allow a full economic analysis of the intervention.

**Table 1:** Schedule of enrolment, interventions and assessments

	**STUDY PERIOD**			
Procedures	Enrolment	Allocation	Post-allocation	
			24 h after birth	Data taken from case notes after discharge
**ENROLLMENT**				
Assessment of eligibility criteria	X	X		
Signed consent form	X			
Randomisation		X		
**INTERVENTIONS**				
Administration of study intervention (misoprostol or oxytocin for augmentation)		X		
**ASSESSMENTS**				
Caesarean Birth		X		
Maternal complications		X	X	X
Fetal / neonatal complications		X	X	X
Serious maternal complications		X	X	X
Cost effectiveness				X
Efficacy of induction process			X	
Assessment of adverse events			X	X
Mother Generated Index	X		X	
Participant Satisfaction Questionnaire			X	

Fetal or neonatal outcomes include neonatal morbidity (meconium-stained liquor; meconium-aspiration syndrome; fetal heart rate abnormality; Apgar score less than seven at five minutes; neonatal intensive care unit admission; seizures; neonatal encephalopathy (as assessed by the Sarnat score)); stillbirth; and perinatal death.

### Sample size calculation

The sample size was estimated based on the primary outcome of caesarean birth assuming a rate of 31% caesarean birth for those receiving oxytocin for augmentation following cervical preparation with oral misoprostol. This is based on published data from a prior trial conducted in this population [14]. A total sample size of 520 women will provide (a) 90% power to detect a reduction in the caesarean rate from 31% to 18.5% (Relative Risk (RR) 0.6), or (b) 80% power to detect a reduction in the caesarean rate from 31 to 20% (RR 0.65) in those women who receive misoprostol (two sided α = 0.05). We estimate that we will need to approach and gain consent from 1,000 women requiring induction of labour for hypertension in order to find 520 who require augmentation after cervical preparation. When augmentation is indicated, consented women will be randomised either to a protocol of continued oral misoprostol (n = 260) or to the standard oxytocin infusion (n = 260).

### Statistical analysis

The trial statistician will provide the Independent Data and Safety Monitoring Committee (IDSMC) with a blinded report every 6 months via email for safety monitoring and will conduct one planned formal interim analysis after 200 women have been recruited. The interim analysis will be for safety and for effectiveness but not for futility; stopping rules will be in accordance with O’Brien-Fleming rules [14] whereby the effective alpha level will be 0.0054 (z = 2.782) for the interim analysis and 0.0492 (z = 1.967) for the final analysis. The IDSMC will have the authority to request further interim analyses if indicated; in the unlikely event this should this occur, revised stopping rules based on the O’Brien-Fleming principles will be calculated according to whether the additional analyses are requested before or after the formal interim analysis at 12 months.

Safety reporting of Serious Adverse Reactions will occur from the period of randomisation until 48 h or discharge, whichever is sooner. Reporting procedures will comply with regulatory authorities in the United Kingdom and India. Trial monitoring will be performed by staff at Gynuity Health Projects on behalf of the sponsor in accordance with the MOLI trial monitoring plan. The research team will share important protocol modifications with relevant parties including investigators, IRBs, trial registries and trial participants as appropriate and in compliance with relevant laws and regulations.

The primary analysis will be performed according to the intention-to-treat principle. The primary outcome measure will be evaluated using binomial regression models, initially unadjusted and then after adjustment for important potential confounding variables and covariates. Binomial regression will be used for binary categorical measures, while ordinal regression will be used for multi-category variables. Count variables will use Poisson regression models and time measures using Cox proportional hazards regression models. All findings will be reported in accordance with the CONSORT guidelines for randomized trials.

All regression models will be run unadjusted (i.e., with treatment group as the only independent/ predictor variable) and then re-run with adjustment for important potential confounder variables/ covariates. These include (but are not necessarily confined to) mother’s age, gestational age, cm dilatation before randomisation, receipt of magnesium sulphate in last 12 h, current anti-hypertensive use, time from hospital admission to randomisation of < 24 h, diastolic blood pressure at enrolment, proteinuria at enrolment, parity, and booked pregnancy. Other potential covariates will include variables found to differ by a clinically significant amount at baseline (post-randomisation) and measures identified as potentially affecting outcome during the study period. Subgroup analysis will be by site of delivery, parity and gestational age. Within the constraints of the O’Brien-Fleming stopping rules, overall statistical significance (across the interim and final analyses) will be set at the conventional 5% level for primary outcomes and p < 0.01 for secondary outcomes. A statistical analysis plan will be completed prior to prior to the first comparative monitoring report to be presented to the IDSMC.

The ISDMC will oversee the safety of participants in the trial. The terms of reference of the ISDMC will be developed separately, based on the principles developed by the DAMOCLES group. The ISDMC, who will have full access to the trial results analysed by allocation, will report directly to the TSC with recommendations about the conduct of the trial as well as whether to continue or stop the trial. The ISDMC will have the following independent voting members: Professor Diana Elbourne (London School of Hygiene and Tropical Medicine), Professor Asmita Muthal-Rathore (Maulana Azad Medical College and Loknayak Hospital, New Delhi), Dr Josh Vogel (Burnet Institute, Melbourne), and Dr Chris Sutton, (The University of Manchester).

## Discussion

Labour induction is one of the most commonly performed obstetric procedures and increasingly common worldwide. In India, almost 1 in 6 deliveries are induced [15]. However, evidence documenting women’s experience and perceptions of the labour induction process and specific cervical ripening and augmentation agents is limited. Most qualitative studies of women’s experience have been conducted in North America and Europe [16, 17]. To help address this gap, our study has employed multiple methodologies – including the utilisation of a mother-generated birth satisfaction index – to improve the recording and understanding of Indian women’s birth priorities and experiences. The use of misoprostol, an oral method for augmentation, has the potential to improve the patient experience by improving women’s mobility during labour. Together with the data from the mother-generated birth satisfaction index, a planned sub-study will undertake semi-structured interviews before the start of the induction and after delivery with a sub-set of women, in order to understand the priorities, experiences and acceptability of induction of labour for women and explore any differences between the two randomized study groups.

We have also planned three additional analyses to help understand the implications of the research findings for the broader health care system. First, as part of the planned qualitative study, our research team will conduct focus group discussions with health care workers at the recruitment sites both before and 4–6 months into the randomised trial. We aim to improve understanding of the feasibility, usability and acceptability of the different induction regimes to health care professionals and to explore potential barriers and solutions for implementing research findings into clinical practice.

Second, we have also planned an economic evaluation to assess patterns and levels of resource utilisation associated with each patient included in the two arms of the randomised trial of induction techniques. The analysis will help identify any variations in resource use (and overall health care expenditure) between the two treatment groups. Finally, we will also undertake a survey of health workers practicing in public health facilities in the two districts where the recruiting hospitals are located to enhance our understanding of provider knowledge, attitudes and practices related to intrapartum uterotonic use. Together these additional analyses will help to contextualise the findings from this randomised trial, improving our understanding of how they can be best implemented and disseminated to improve Indian women’s birth experience during induction of labour.

## Data Availability

The data from this study will be confidential until the database is closed at the end of the study. Following this the study investigators will have exclusive access to the data until the publication of the results in a journal. Once this has happened, the database will be open to other researchers upon request. Open access databases will also be sought so as to maximise the availability of our research data with as few restrictions as possible, in line with MRC and Wellcome Trust policy. The consent form will include a clause for the woman to give permission for her anonymous data to be used for future research studies.

## Abbreviations

ARM: Artificial rupture of membranes
IDSMC: Independent Data Safety Monitoring Committee
RR: Relative Risk
